# Comparison of Quality and Sensory Characteristics of Spent Hen and Broiler in South Korea

**DOI:** 10.3390/ani11092565

**Published:** 2021-08-31

**Authors:** Sol-Hee Lee, Hack-Youn Kim

**Affiliations:** Department of Animal Resources Science, Kongju National University, Yesan-Gun 32439, Korea; chzh73@naver.com

**Keywords:** spent hen, broiler, breast, thigh, quality and sensory characteristics

## Abstract

**Simple Summary:**

Spent hen and broiler have clear differences according to consumption methods. The pH, color, cooking yield, water holding capacity (WHC), and shear force of carcass characteristics and the electronic nose and electronic tongue of sensory characteristics were compared in order to recruit discarded spent hens. As a result, spent hen had a lower cooking yield and WHC compared to broiler and thus presented a higher shear force. In addition, broiler showed higher umami values than spent hen in the electronic tongue. Accordingly, it is essential to use additives with a high water holding capacity to process spent hen meat.

**Abstract:**

This study was conducted to compare the quality and sensory characteristics of spent hen and broiler in South Korea. The carcasses of spent hens and broilers that had been slaughtered 24 h before were used. The cooking yield and water holding capacity of broiler was significantly higher than that of spent hen (*p* < 0.05). The pH of broiler thigh was significantly higher than that of the other treatments (*p* < 0.05), while on the other hand spent hen breast had a lower value than other treatments (*p* < 0.05). The redness of the thigh of both types was more than that of the breast (*p* < 0.05). In contrast, the yellowness of the breast of the two types was more than that of the thigh of both types. The changes in organoleptic characteristics of broiler was higher than that of spent hen, and the aroma patterns detected using the electronic nose were markedly different in terms of the type of meat. Spent hens are generally considered to have lower consumption rates than broilers because they have a lower taste and aroma. Therefore, the results of this study suggest that processing or additives are required in the distribution method of spent hens.

## 1. Introduction

Chickens are classified into spent hen and broiler in South Korea. Spent hens refer to hens with an increased feed demand and reduced laying efficiency due to old age [1]. Because these are old spent hens that have stopped laying eggs, the meat has a high collagen content, resulting in a tough texture [2,3]. Park et al. [4] reported that spent hens have a high concentration of insoluble protein and that their meat contains tissues that are difficult to process by heating. On the other hand, broilers are bred for meat, and their meat retains its elasticity even after heating, which makes it well-suited to consumer preferences [5]. Compared to spent hen, broiler meat contains at least 20% less unsaturated fatty acids, which leads to better storage stability [6].

According to the Korean Statistical Information Service, the breeding numbers of spent hens and broilers were 70 million and 93 million, respectively, in 2019; however, the number of slaughters showed a large difference, with 200 spent hens and 70 million broilers in the same year [7]. As a result, spent hens show difficulties in terms of the consumption of spent hen at the end of their economic life cycle, and they are typically exported to other countries or sold at a cheap price [8]. On the other hand, broilers steadily increased from 9.62 kg/person in 2009 to 14.80 kg/person in 2019 [9]. As the consumption patterns between the two breeds are in stark contrast, it is thought to be important to understand the appropriate processing method through the analysis of the two breeds.

The analysis of the sensory properties of ingredients is important because it is related to the type of meat, genetics, and breeding [10]. As such, there is a long history of training tasting panels and conducting sensory evaluations to analyze preferences, including taste, flavor, and texture, as part of the product development cycle [11]. However, it is difficult to recruit and train people to participate in a sensory evaluation panel, and thus the application of instrumental measurements such as shear force, the electronic tongue, and the electronic nose can provide reliable data on the eating quality of food products [12,13].

Although there have been several reports on spent hens and broilers, most studies have focused on the individual properties of each type of meat, rather than comparing the two types. The aim of the present study was to compare the physicochemical and sensory characteristics of the two types of meat and analyze the meat quality and manufacture of processed meat products, in order to provide basic data for future experiments on chicken meat.

## 2. Materials and Methods

### 2.1. Preparation of Spent Hens and Broilers

Spent hen was used for the 75-week-old hy-line brown breed, and broiler was used for the 4-week-old one. Refrigerated spent hen and broiler from 24 h after slaughter were purchased from the local slaughterhouse. Spent hens and broilers were bred at different farms depending on the species, in compliance with the National Research Council (NRC) specification standards, and were slaughtered and used at the same slaughterhouse (Chungnam, Korea). The purchased samples were placed in an ice box and transferred to a refrigerated state to conduct the experiment. The experiment was performed in a total of 3 repetitions, and the number of individuals per repetition was 15 animals. The sample was directly deboned to obtain the breast and thigh, which were stored at 5 °C until further use. All of the experimental samples did not exceed a week at any one time, and in the case of the electronic nose and electronic tongue, the samples were frozen and used.

During the trial, the slaughter of samples was carried out in a slaughterhouse licensed by the South Korea state; therefore, approval by the ethics committee was not considered necessary.

### 2.2. pH

Uncooked meat was homogenized with distilled water (DW) in a 1:4 ratio using Ultraturax (HMZ-20DN, 82 Pooglim Tech, Seoul, South Korea) at 6780× *g*, and turbidity was measured using a pH meter (S220, Mettler-Toledo, Greifensee, Switzerland).

### 2.3. Color

Lean meat was cut into a form of approximately 2.0 cm (height) and measured using a colorimeter (CR 210, Minolta, Tokyo, Japan) using CIE L* (lightness), CIE a* (redness), and CIE b* (yellowness) values. The color experiment was tested in a total of 5 repetitions.

### 2.4. Cooking Yield

The cooking yield of the samples was measured before and after cooking. The meat was cooked for 1 h in a chamber set to 75 °C, and the samples were then cooled for 20 min at room temperature (25 °C). The cooking yield was calculated using the following equation:

Cooking yield (%) = weight after cooking (g)/weight before cooking (g) × 100(1)

### 2.5. Water Holding Capacity

The water holding capacity (WHC) of chicken meat was measured using the filter paper-press method [14]. First, 0.3 g of meat was weighed in a filter paper (Whatman No. 2; GE Healthcare life sciences Whatman, Chicago, IL, USA) and compressed for 3 min using a filter-press device. After drying the filter paper with the sample in a desiccator (sensor MT-10S, MT Precision, Tokyo, Japan) for approximately 1 day, WHC was calculated using the following equation:

WHC (%) = meat area (mm^2^)/exuded water area (mm^2^) × 100 (2)

### 2.6. Shear Force

The test of the samples was measured using the method of Park et al. [14]. The shear force of the cooked meat samples was measured using a texture profile analysis instrument (TA1, Lloyd, Largo, FL, USA). The cooked meat was cut in the same direction as the muscle and measured at right angles to the direction of the muscle. The conditions of measurement were as follows: v-vlade, head speed, 2.0 mm/s; distance, 2.0 mm; force, 5 g; and the measured value, kg.

### 2.7. Electronic Tongue

The E-tongue was based on the modified method of Lee et al. [15]. Eight grams of chicken meat were weighed and homogenized for 1 min with 32 mL of DW using a homogenizer (AM-5, Nissei, Anjo, Japan). The homogenate was filtered using filter paper (Whatman No. 1, GE Healthcare, Chicago, IL, USA), and the filtrate was diluted 100 times in a glass container. Changes in organoleptic characteristics were measured using an electronic tongue system (Astree 5, Alpha MOS, Toulouse, France). Seven sensors, namely CTS, NMS, AHS, PKS, SCS, ANS, and CPS, were used. AHS, NMS, and CTS indicate the sourness, umami, and saltiness sensors, respectively, while PKS, CPS, ANS, and SCS are general-purpose sensors.

### 2.8. Electronic Nose

The test of the samples was measured using the modified method of Kang et al. [16]. The principal component analysis (PCA) of the chicken meat was performed using an electronic nose system (Heracles-II-e-nose, Alpha MOS, Toulouse, France). Five grams of each ground sample were placed in vials which were then capped. The samples were incubated for 20 min at 80 °C in a controlled thermostatic agitator. Subsequently, the samples in the vials were collected under the following conditions: headspace injection of 2.0 mL, injection speed of 200 µL/s, and injection temperature of 200 °C. MXT-5 and MXT-1701 were mounted, and the detector temperature was maintained at 260 °C while measuring and analyzing the sample aroma pattern using PCA (Alpha MOS, Toulouse, France).

### 2.9. Statistical Analysis

Statistical analysis was conducted with the experimental results for two types of birds (spent hen and broiler) and two types of muscle (breast and thigh). The cooking yield, WHC, shear force, pH, and color were analyzed using a mixed model in SAS (version 9.3; SAS Institute, Cary, NC, USA), and the results are expressed as mean ± standard deviation. Significant differences (*p* < 0.05) among the mean values were determined using an analysis of variance and Duncan’s multiple range test. The correlations between WHC and cooking yield and WHC and shear force were determined using the CORR function in the SAS analysis.

## 3. Results and Discussion

### 3.1. pH and Color

The pH and color of the uncooked samples of the breast and thigh of spent hens and broilers were measured (Table 1). The pH was significantly lower in SB (*p* < 0.05) and significantly higher in BT (*p* < 0.05). Because meat shows an isoelectric pH of 5.2–5.6, the positive and negative charges are close to neutral in this interval [17]. This means that there is less space to store water inside meat proteins; therefore, the WHC is lowest at the isoelectric point [18]. Thus, the study is thought to have affected the findings, which were that the cooking yield also decreased as the pH approached the isoelectric point. This is consistent with a study by Park et al. [19], in which the pH in the breast and thigh from chickens of different ages were compared and a higher pH was observed in the thigh than in the breast. In addition, the spent hen showed a significantly lower pH than the broiler, which is thought to be due to the decrease in the water content in the muscles with increasing age. Beak [20] et al. showed the same results as in this study, saying that the pH decreased as the age of the laying hen increased.

In addition to pH, Table 1 shows the results for the color of the breast and thigh of the spent hen and broiler. Color is an important factor because it has a major, direct impact on consumers’ purchasing rates and preferences [21]. Lightness was significantly higher in BT than in SB and BB, which can be explained by the higher accumulation of the blood pigment myoglobin in thigh meat than in breast meat, which has a low exercise capacity. von Lengerken et al. [22] previously reported that the majority of muscle fibers in the breast were white fibers, while the majority of muscle fibers in the thigh were red fibers. Redness was significantly higher in ST and BT than in SB and BB, which was also thought to be due to myoglobin. This is similar to the results of a study by Kadıoğlu et al. [23], who studied the color of spent chicken meat and reported more redness in the thigh than in the breast. Yellowness was significantly higher in the SB group than in the other groups, which was similar to the findings of Baek et al. [20], which showed that yellowness increased with age.

### 3.2. Cooking Yield and Water Holding Capacity

**Figure 1:**
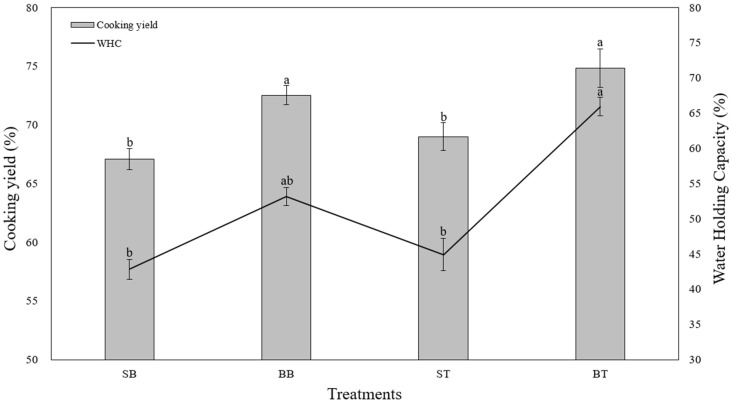
Cooking yield and WHC calculated from the breast and thigh of spent hens and broilers. Standard deviations are expressed on bars. a,b in the same test with different letters indicates that the results are significantly different (*p* < 0.05). SB, spent hens breast; BB, broilers breast; ST, spent hens thigh; BT, broilers thigh.

Figure 1 shows the cooking yield and WHC of the breast and thigh of spent hens and broilers. The cooking yields of the breast of spent hens (SB) and the thigh of spent hens (ST) were significantly lower than those of the breast of broilers (BB) and thigh of broilers (BT; *p* < 0.05). As the structure of the muscle becomes smaller during maturation, the muscle has less space to retain, that is, the WHC is lowered [24]. Therefore, in this study, it is also judged to have a low cooking yield in spent hen. Additionally, regardless of the two breeds, between the breast and thigh there are different values (*p* > 0.05). This is due to the higher fat content in the thigh, which is red meat, compared to the breast, thereby resulting in a high water-binding capacity. This is consistent with a previous report by Lee et al. [25] who found that a higher intramuscular fat content was associated with a higher cooking yield. van der Sman [26] reported that the application of a physical force, such as heat, caused protein degradation resulting in the release of water from inside the muscle fibers. Therefore, the chicken breast, which has a high protein content, showed a higher volume of water loss compared to the thigh.

WHC refers to the force with which meat holds onto water throughout all processes including transport, storage, and mechanical processes such as cutting, heating, and grinding [23]. WHC and the cooking yield showed similar patterns; WHC was significantly higher for BT than for SB and ST (*p* < 0.05). Although it is the same breast, it seems that spent hen lacks the ability to bind moisture compared to broiler under heating stress. A study found that the water absorption capacity of myofibrillar protein in chicken breast was superior to that of thigh [27]. Therefore, it seems that additional research is needed to increase the moisture content by using any moisture treatment. Cao et al. [28] reported an increase in WHC after an ultrasound-assisted enzymatic treatment. In another study, WHC was improved physico-chemically using bromelain enzyme [29].

### 3.3. Shear Force

Table 2 shows the shear force measurements for the breast and thigh of spent hens and broilers. SB showed a higher shear force than the other groups and a significantly higher shear force than BT (*p* < 0.05), due to the loss of moisture from myofibrils during cooking, which leads to a higher shear force [16]. This corroborates the results in the present study, which also demonstrated a decreasing shear force with higher WHC. This was similar to a report by Choe et al. [30] who demonstrated that breast meat was harder than thigh meat in Korean native chickens. In addition, Baéza et al. [31] reported that the fiber cross-sectional area hypertrophies as age increases, so it seems that the shear force of spent hens is high. Based on the findings in the present study, rather than using and selling SB, which has a high shear force, in its raw state, it would be better distributed as a processed product.

### 3.4. Changes in Organoleptic Characteristics Detected Using Electronic Tongue

**Figure 2:**
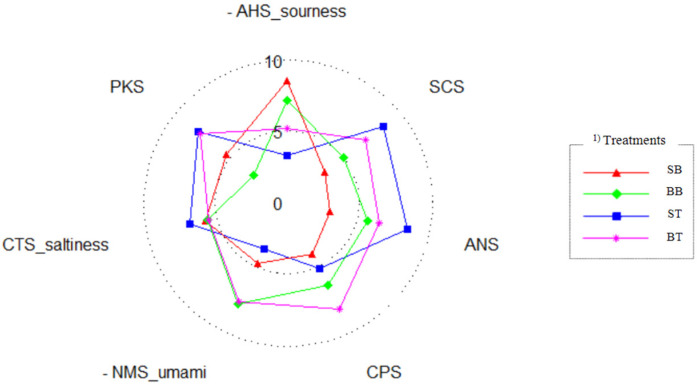
Changes in organoleptic characteristics detected using an electronic tongue on the breast and thigh of spent hens and broilers. SB, spent hens breast; BB, broilers breast; ST, spent hens thigh; BT, broilers thigh.

Figure 2 shows the taste scores of the breast and thigh of spent hens and broilers which were analyzed using an electronic tongue. Sourness was the highest for SB at 8.5 and the lowest for ST at 3.2. Sourness was higher in the breast than in the thigh, irrespective of the type of chicken. Sourness in meat has been reported to be related to phenylalanine and isoleucine [32], and Chae et al. [33] reported that phenylalanine and isoleucine levels were higher in the breast than in the thigh, supporting the findings of the present study. The saltiness was 6.9 in ST and 5.6–5.8 in the other meats. Umami has been reported to be the fifth taste that can be perceived by humans, corresponding to the taste of monosodium glutamate and 5-ribonucleotides [34,35]. The umami score in BB and BT was 7.85, which was higher than the mean umami score in SB and ST (3.6). Glutamic acid is a free amino acid with the greatest effect on both saltiness and umami and has been reported to be present at higher levels in the thigh than in the breast [36]. Threonine, asparagine, and glycine have also been reported to affect the customer’s preference, which is consistent with studies showing lower concentrations of these amino acids with increasing age [37]. The results of the present study showed that the thigh and breast meats were differentiated by sourness, whereas spent hens and broilers were differentiated by umami. Therefore, it would be advisable to add seasoning when processing spent hen meat to enhance its weak umami taste.

### 3.5. Principal Component Analysis by Electronic Nose

**Figure 3:**
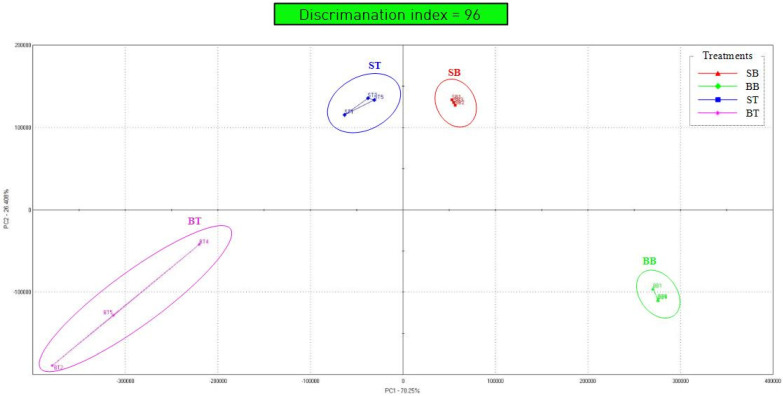
Principal component analysis using an electronic nose on the breast and thigh of spent hens and broilers. SB, spent hens breast; BB, broilers breast; ST, spent hens thigh; BT, broilers thigh.

Figure 3 shows a comparison of aroma patterns between the breast and thigh of spent hens and broilers using PCA on data from an electronic nose. Previously, aroma patterns were determined using gas chromatography or gas chromatography-mass spectrometry. However, because these methods take a long time, electronic nose analysis is widely used at present [38]. The *X*-axis showed the largest variance, differentiating ST and BT (left of 0) from SB and BB (right of 0). The *Y*-axis showed the second largest variance, clearly differentiating SB and ST (above 0) from BT and BB (below 0). Compared to the electronic analysis, the *X*-axis was surmised to be affected by sourness, which distinguished between the breast and thigh, irrespective of the chicken type. Because the *Y*-axis distinguished between spent hens and broilers, it appeared to be affected by umami. In the graph, the breast formed small triangles while the thigh formed large triangles, which indicated that thigh meat included a large amount of connective tissue and that there was a large variance in aroma between different parts of the thigh. Overall, there was a clear separation of SB, ST, BB, and BT in the graph, indicating that each group had distinct aromas.

## 4. Conclusions

As a result of this study, through an analysis of the quality and sensory characteristics of spent hens and broilers, we concluded the following. The color was the only difference between the breast and thigh; there was no other visual difference. The cooking yield was significantly different between the spent hens and broilers. The WHC was highest in the thigh of broilers when compared to other samples, but not significantly higher than the breast of broilers. The shear force was significantly higher in the breast of spent hens than in the thigh of broilers. Spent hens and broilers were significantly different according to the data collected using the electronic tongue and electronic nose. Consequently, there was a great difference in the parameters that affect palatability such as the cooking yield, WHC, and shear force. Thus, spent hen has a lower taste value than broiler, and processing spent hen is considered to require a pulverized meat product or flavor enhancer such as sodium glutamate, mono-sodium L-glutamate, etc.

## Figures and Tables

**Table 1:** Breast and thigh muscle pH and color in spent hens and broilers.

Traits		Treatment ^1^			
		SB	BB	ST	BT
pH		5.64 ± 0.06 ^c^	6.43 ± 0.03 ^b^	6.37 ± 0.06 ^b^	6.83 ± 0.06 ^a^
Color	CIE L^*^	55.50 ± 0.79 ^a^	54.63 ± 0.19 ^a^	50.86 ± 0.61 ^b^	52.94 ± 0.38 ^ab^
	CIE a^*^	4.27 ± 0.46 ^b^	5.82 ± 0.15 ^b^	7.23 ± 0.22 ^a^	8.08 ± 0.22 ^a^
	CIE b^*^	6.12 ± 0.15 ^a^	5.68 ± 0.29 ^b^	4.30 ± 0.83 ^bc^	3.46 ± 0.27 ^c^

All values are represented as mean ± standard deviation. ^a–c^ in the same row with different letters indicates that the results are significantly different (*p* < 0.05). ^1^ SB, spent hens breast; BB, broilers breast; ST, spent hens thigh; BT, broilers thigh.

**Table 2:** Shear force in breast and thigh muscles from spent hens and broilers.

Traits	Treatment ^1^			
	SB	BB	ST	BT
Shear force (kg)	3.41 ± 0.17 ^a^	3.29 ± 0.24 ^ab^	2.69 ± 0.20 ^ab^	2.54 ± 0.28 ^b^

All values are represented as the mean ± standard deviation. ^a,b^ in the same row with different letters indicates that the results are significantly different (*p* < 0.05). ^1^ SB, spent hens breast; BB, broilers breast; ST, spent hens thigh; BT, broilers thigh.

## Data Availability

Not applicable.
